# Diversity and Expression of MicroRNAs in the Filarial Parasite, *Brugia malayi*

**DOI:** 10.1371/journal.pone.0096498

**Published:** 2014-05-13

**Authors:** Catherine B. Poole, Weifeng Gu, Sanjay Kumar, Jingmin Jin, Paul J. Davis, David Bauche, Larry A. McReynolds

**Affiliations:** 1 Division of RNA Biology, New England Biolabs, Ipswich, Massachusetts, United States of America; 2 Division of Parasitology, New England Biolabs, Ipswich, Massachusetts, United States of America; 3 Program in Molecular Medicine, University of Massachusetts Medical School, Worcester, Massachusetts, United States of America; 4 Cancer Research Center of Lyon, Lyon, France; George Washington University School of Medicine and Health Sciences, United States of America

## Abstract

Human filarial parasites infect an estimated 120 million people in 80 countries worldwide causing blindness and the gross disfigurement of limbs and genitals. An understanding of RNA-mediated regulatory pathways in these parasites may open new avenues for treatment. Toward this goal, small RNAs from *Brugia malayi* adult females, males and microfilariae were cloned for deep-sequencing. From ∼30 million sequencing reads, 145 miRNAs were identified in the *B. malayi* genome. Some microRNAs were validated using the p19 RNA binding protein and qPCR. *B. malayi* miRNAs segregate into 99 families each defined by a unique seed sequence. Sixty-one of the miRNA families are highly conserved with homologues in arthropods, vertebrates and helminths. Of those miRNAs not highly conserved, homologues of 20 *B. malayi* miRNA families were found in vertebrates. Nine *B. malayi* miRNA families appear to be filarial-specific as orthologues were not found in other organisms. The miR-2 family is the largest in *B. malayi* with 11 members. Analysis of the sequences shows that six members result from a recent expansion of the family. Library comparisons found that 1/3 of the *B. malayi* miRNAs are differentially expressed. For example, miR-71 is 5–7X more highly expressed in microfilariae than adults. Studies suggest that in *C.elegans*, miR-71 may enhance longevity by targeting the DAF-2 pathway. Characterization of *B. malayi* miRNAs and their targets will enhance our understanding of their regulatory pathways in filariads and aid in the search for novel therapeutics.

## Introduction

The lymphatic filarial parasites *Brugia malayi*, *Brugia timori* and *Wuchereria bancrofti* infect an estimated 120 million people in 80 countries worldwide [1]. They are transmitted by mosquitos harboring infective third stage larvae (L3s) that upon entering the vertebrate host, molt to L4s which mature to adulthood over the course of 6–12 months [2]. Adult parasites settle in the lymphatic vessels and mate producing microfilariae (mf). The mf can survive for up to a year migrating throughout the peripheral circulation waiting to be ingested by a mosquito during a blood meal [3].

Lymphatic filarial infections are characterized by recurrent fevers, painful adenolymphangitis and elephantiasis [4]. Although not considered fatal, the morbidity caused by filarial infections greatly impedes socio-economic development in affected communities [5]. Diethylcarbamazine (DEC), ivermectin and albendazole are the drugs commonly used to treat lymphatic filarial infections. All three kill microfilariae but only DEC exhibits limited efficacy against adult parasites [6]. The recent appearance of drug resistance against ivermectin [7] and the lack of good macrofilariacides necessitate the development of new approaches for combating this debilitating disease. The complex filarial life cycle and the inability to genetically manipulate the parasite make biological studies difficult. Recently, molecular approaches including EST and genome sequencing of *B. malayi* [8], [9] and related filarids [10], [11] have greatly expanded our knowledge of the expression and evolution of filarial genes. We have extended this approach by cloning and high-throughput sequencing of *B. malayi* small RNAs. An understanding of RNA-mediated regulatory pathways in filarial parasites may open new avenues for treatment. For example, identification of filarial-specific components of small RNA pathways or miRNAs may be leveraged for the development of novel anti-filarial agents.

*Caenorhabditis elegans lin-4* was the first gene discovered to encode a small RNA and demonstrated to post-transcriptionally regulate LIN-14 protein levels by binding to complementary sequences in the 3′UTR of its mRNA [12], [13]. MicroRNAs function through ARGONAUTE proteins, a component of the RNA induced silencing complex (RISC). In general, microRNAs guide RISC to sequences in the 3′ UTR of mRNAs complementary to nucleotides 2–7 of the miRNA known as the “seed” sequence [14], [15], [16] however, microRNA sequence outside of the seed can compensate for weak or imperfect seed pairing [15], [17], [18], [19], [20]. Once bound, mRNA stability and translational suppression is mediated through the interaction of miRNA-RISC with members of the GW182 protein family [21], [22].

It is now known that miRNAs are ancient in origin. They are found in an evolutionarily diverse assortment of organisms ranging from sponges to vertebrates [23], [24]. MicroRNAs in the free-living nematode, *C. elegans* are well characterized [25], [26], [27], [28], [29], [30], [31] but little is known about them in parasitic nematodes. Our initial work to characterize small RNAs in *B. malayi* identified 32 miRNAs using bioinformatic and cloning approaches [32]. *C. elegans* (100 Mb) and *B. malayi* (90–95 Mb) likely encode similar numbers of miRNAs given that their genome sizes are roughly equivalent [9]. The goal of this present study is a more comprehensive identification of miRNAs in *B. malayi* and to compare the findings to what is known in *B. pahangi* [33], a closely related filarial parasite and *C. elegans*. This study forms the background for understanding miRNA function in light of the complex *B. malayi* lifecycle and can be used as the basis for designing anti-miRNA compounds that are lethal to the parasite.

## Results & Discussion

### Library Overview

This publication is an in depth characterization of the diversity and expression of miRNAs from different stages of the human filarial parasite, *B. malayi*. Our initial publication [32] describes our analysis of only a few hundred small RNA reads while in this publication, we report the analysis of ∼30 million reads for miRNAs from 3 different stages of the parasite.

Five small RNA libraries were prepared from *B. malayi* males, females and mf using 3 different protocols (Table 1) that distinguish between differences in the phosphorylation states of small RNAs [34], [35], [36] and to minimize the prevalence of degradation products. The male, female and one mf library were prepared with calf intestinal phosphatase, (CIP) and T4 polynucleotide kinase. Treatment with CIP followed by T4 polynucleotide kinase enabled all small RNA populations including RNA degradation products with 5′OH groups to ligate to the 5′ linker. Although ∼71–74% of the reads from the CIP libraries were ≥17 nt long and an exact match to the *B. malayi* genome, ∼6–11% of reads matched the 18S rRNA gene indicating significant levels of degradation in these libraries (Table 1). To address this problem, two additional libraries (DIR and TAP) were prepared from the same mf RNA sample. These libraries were constructed using microfilariae because they are abundant and easier to obtain than adult parasites. In the DIR library, RNA is directly ligated to the 5′ linker without pretreatment. Using this method, only small RNA populations, including endogenous miRNAs, with a single 5′ phosphate and 3′ OH will ligate to the linkers. In the mf TAP library, caps and polyphosphates on small RNA populations are digested to a single 5′ phosphate using Tobacco Acid Pyrophosphatase prior to ligation with the 5′ linker. Both the DIR and TAP protocols minimize ligation of degradation products to the 5′ linker as demonstrated by the 45X reduction in the reads matching the *B. malayi* 18S rRNA gene from ∼11% in the CIP libraries to 0.25% in the DIR and TAP libraries (Table 1).

**Table 1:** *B. malayi* Small RNA Library Overview.

	Male CIP	Female CIP	mf CIP	mf DIR	mf TAP
Total Reads^a^	3575204	3605031	3791460	10468773	8906366
≥17 nt^b^	2880756	2807748	3172516	7501197	6782572
≥17 nt (%)^c^	80.6	77.9	83.7	71.6	76.1
Genome hits^d^	2060697	2002201	2350335	3738980	3849562
Genome hits (%)^e^	71.5	71.3	74.1	49.8	56.7
18S rRNA hits^f^	373092	237844	393830	20054	16259
18S rRNA hits (%)^g^	10.4	6.6	10.4	0.2	0.2

a total number of reads from each library.

b number of reads from each library that are ≥17 nt long.

c ≥17 nt reads/total reads X 100.

d number of reads ≥17 nt long that are an exact match to the *B. malayi* genome.

e genome hits/≥17 nt reads X 100.

f number of 18S rRNA reads in each library (Genbank accession no. AF036588).

g 18S rRNA reads/total reads X 100.

The TAP and DIR libraries were constructed from the same mf RNA sample and as expected, the relative abundance of miRNA reads from these two libraries is very similar: 98% of the standardized miRNA reads are within two fold (Tables S1 & S2). However, comparison of miRNA reads from libraries prepared from separate RNA samples using different methods such as the CIP and DIR mf libraries, resulted in only 52% of the miRNA reads being within two fold (Tables S1 & S2). The comparison was made between miRNAs that represented more then 0.01% of total miRNA reads in a library.

Only 11 miRNAs were identified in the DIR and/or TAP mf libraries that were not found in the CIP mf library (Table S1). All these miRNAs were present at very low read numbers (<10) and were likely observed because of the increased depth at which the DIR and TAP libraries were sequenced compared to the CIP libraries (Table 1).

These libraries consist of a variety of overlapping as well as unique small RNA populations. The DIR library consists primarily of small RNAs processed by Dicer such as miRNAs [37], [38] whereas the TAP library consists of capped and polyphosphorylated small RNA populations in addition to Dicer products [38].

### MicroRNA Discovery and Family Assignment

**Figure 1:**
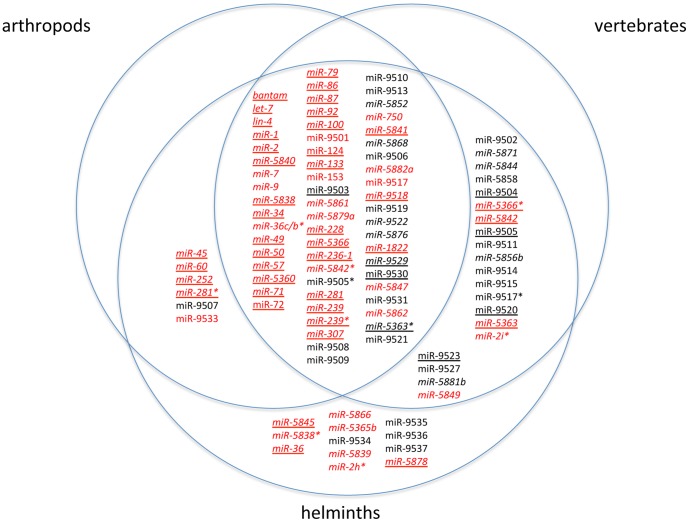
Conservation of *B. malayi* miRNAs families. Venn diagram depicting the conservation of the 99 miRNA families among arthropods, vertebrates and helminths. Homologues were identified by searching miRBase (versions 15–18) for miRNAs with a minimum of 6 contiguous nt between positions 1–7 or that exhibit ≥70% overall identity. *B. malayi* miRNAs represented by ≥100 reads in one of the libraries are highlighted in red. MicroRNAs represented by <100 reads are black (Tables S3 and S4). *B. malayi* families with a homologue in *B. pahangi* (Table S9) are highlighted with italics. *B. malayi* families with a homologue in *C. elegans* (Table S7) or other helminth are underlined.

A total of 145 miRNAs (from 129 hairpin sequences) were identified in the *B. malayi* small RNA libraries. These include 96 prevalent miRNAs (≥100 reads in one of the 5 libraries, Table S3) and 49 rare (<100 reads, Table S4). Thirty-two of the prevalent miRNAs were described previously [32]. The *B. malayi* miRNAs segregate into 99 families each defined by a unique seed sequence ([16], [39], Figure 1). Sixty-six miRNAs segregate into 20 families of paralogues based on nucleotide identity in the 5′ seed region or by global identity of ≥70% ([40], Tables 2 and S5). The remaining 79 families consist of a single miRNA.

**Table 2:** Comparison of miRNA Families in *B. malayi* and *C. elegans*.

nematode	total miRNAs	total hairpins	afamilies of paralogues	bsingleton families	ctotal families	dshared families	eunique families
*B.malayi*	145	129	20	79	99	48	51
*C.elegans*	195	175	40	61	101	48	53

a MicroRNAs sharing a minimum of 6 contiguous nt between positions 1–7 at the 5′ end or that exhibit ≥70% identity overall in the absence of a conserved 5′ end were grouped into families of paralogues. In *B. malayi*, 66 miRNAs were grouped into 20 families (Table S5). In *C. elegans*, 134 miRNAs were grouped into 40 families (Table S6).

b The remaining miRNAs in each nematode that do not fall within a paralogous group are considered individual families consisting of a single member.

c Total number of families  =  number of paralogous families + number of singleton families.

d 48 miRNA families are shared between *B. malayi* (75 miRNAs) and *C. elegans* (113 miRNAs). See Table S7 for details.

e Number of families in a nematode with a seed sequence that is not found in the other nematode.

For comparison with *B. malayi*, 195 *C. elegans* miRNAs were downloaded from miRBase release 15 and analyzed using the same criteria. The *C. elegans* miRNAs segregate into a similar number of families (101) compared to *B. malayi* (99) consisting of 40 families of paralogues; twice the number found in *B. malayi* (Tables 2 and S6). About half of these *B. malayi* miRNA families, 48 families (representing 75 miRNAs) are also found in *C. elegans* (Tables 2 and S7).

Approximately 25% fewer *B. malayi* miRNAs have been identified than have been found in *C. elegans* (195, miRBase release 15), a nematode with a similar genome size [9], [41]. There are several likely reasons for this difference. MiRDeep is reported to identify 70–90% of miRNAs depending on the data set being mined [42]. Because of its requirement for genomic sequence, miRNAs were probably missed because the *B. malayi* genome is only 75–80% complete [9]. We were able to identify 5 highly conserved miRNAs by searching the small RNA libraries directly. For example, we have been unable to identify the highly conserved miR-1 presumably because its DNA sequence is missing from the genome [32] but it was easily identified by directly searching the small RNA libraries with the *C. elegans* miR-1 sequence. It is one of the most abundant miRNAs to be identified and represents 8.9–11.5% of the total miRNAs in the adult and mf libraries (Tables S1–S3). A current version of the *B. malayi* genome is being curated by WormBase (www.wormbase.org). As the remainder of the *B. malayi* genome sequence becomes available, more miRNAs will likely be identified in these libraries as well as in small RNA libraries of other developmental stages.

The *B. malayi* miRNAs were also compared to miRNAs identified in *B. pahangi* [33]. Fifty-five of the 99 *B. malayi* miRNA families are conserved in *B. pahangi* based on nucleotide identity in the 5′ seed region or by global identity of ≥70% ([40]; Tables 3, S8 and S9). This low level of homology between the two closely related *Brugia* species is likely explained by the methodology used to identify the *B. pahangi* miRNAs as well as the fact that different life cycle stages were sequenced from *B. pahangi* then in this work [33]. The *B. pahangi* miRNAs were identified based on *B. malayi* genome and any miRNAs with a single nt mismatch to the *B. malayi* genome were discarded [33].

**Table 3:** Comparison of miRNA Families in *B. malayi* and *B. pahangi*.

nematode	total miRNAs	total hairpins	afamilies of paralogues	bsingleton families	ctotal families	dshared families	eunique families
*B.malayi*	145	129	20	79	99	55	44
*B.pahangi*	134	ref [33]	14	66	80	55	25

a MicroRNAs sharing a minimum of 6 contiguous nt between positions 1–7 at the 5′ end or that exhibit ≥70% identity overall in the absence of a conserved 5′ end were grouped into families of paralogues. In *B. malayi*, 66 miRNAs were grouped into 20 families (Table S5). In *B. pahangi*, 38 miRNAs were grouped into 14 families (Table S8).

b The remaining miRNAs in each nematode that do not fall within a paralogous group are considered individual families consisting of a single member.

c Total number of families  =  number of paralogous families + number of singleton families.

d 55 families are shared between *B. malayi* (80 miRNAs) and *B. pahangi* (77 miRNAs). See Table S9 for details.

e Number of families in a nematode with a seed sequence that is not found in the other nematode.

### MicroRNA validation using p19 and qPCR

Two different methods, other then sequencing, were used to measure the relative abundance of four miRNAs from three different stages of the parasite. The p19 protein from the Carnation Italian ringspot virus binds 20–23 nucleotide long dsRNA molecules in a size dependent, sequence independent manner. This protein does not bind ssRNA. Using a labeled RNA probe complementary to a specific miRNA, the p19 protein can isolate miRNA:RNA probe duplexes in a million fold excess of total RNA. Using a radioactive probe, this method can detect miRNAs in the sub-picogram range [43]. The selective binding properties of p19 have also been used in conjunction with nanopore [44] and electrochemical detection [45] to measure very low levels of miRNAs.

The p19 detection and qPCR were used to measure relative abundance of four miRNAs in males, females and mf. The miRNAs were chosen to represent a range of expression and stage specificity. The very abundant miR-71 represents 27% of all miRNA reads in the CIP mf library while miR-5842* only represents 0.016% of the reads in the mf CIP library (Table S2). For stage specific expression, miR-36c* was chosen because it is preferentially expressed in females (0.469%) compared to males (0.018%) and mf (0.001%; Table 4).

**Table 4:** Stage-Enriched Expression of *B. malayi* miRNAs^a^.

	CIP Library		
miRNA	Male	Female	mf
let-7	**2.200**	**0.428**	0.001
lin-4	**0.940**	**0.102**	0.002
miR-133	**0.079**	**0.009**	0.001
miR-236-1	**3.780**	**2.227**	0.036
miR-239	**0.668**	**0.238**	0.031
miR-239*	**0.043**	0.004	0.001
miR-283	**3.358**	**0.460**	0.006
*miR-2a*	*0.282*	*0.264*	***2.030***
miR-2b	**0.062**	**0.472**	0.004
miR-2b-2*	**0.117**	**1.733**	0.022
miR-2e	**1.521**	**0.231**	0.001
miR-2h*	0.003	**0.096**	0.000
mir-2i*	0.007	**0.062**	0.009
miR-36a	**0.404**	**8.231**	0.012
miR-36b	**0.057**	**0.279**	0.002
miR-36c	**0.140**	**0.782**	0.003
miR-36c/b*	**0.018**	**0.469**	0.001
miR-36d	**0.006**	**0.027**	0.000
miR-36d*	0.008	**0.107**	0.004
miR-5360	**0.562**	**1.014**	0.048
miR-5361	**0.159**	**0.372**	0.014
miR-5363	**0.237**	0.033	0.009
miR-5364	**7.582**	**4.744**	0.121
miR-5365b	**0.146**	0.008	0.017
miR-5366*	*0.000*	*0.003*	***0.019***
miR-57	**0.703**	**0.105**	0.022
miR-5838	**0.676**	**0.016**	0.001
miR-5838*	**0.254**	**0.008**	0.000
miR-5839	**0.016**	**0.053**	0.000
miR-5840	**0.031**	**0.224**	0.000
miR-5841	**0.542**	**0.137**	0.013
miR-5847	**0.005**	0.000	0.000
*miR-71*	*5.933*	*3.803*	***27.427***
miR-84	**19.144**	**8.641**	0.009
*miR-92*	*0.040*	*0.047*	***0.268***

a The prevalent miRNAs (Table S3) that exhibit a minimum 5X increase in expression (bold) between adult and mf CIP libraries are shown. When 2 miRNAs are excised from opposite arms of the same hairpin, a star (*) after the name generally denotes the less frequent form [25], [50]. The values from Table S2 represent the % of the total miRNA population in each CIP library. MicroRNAs predominately expressed in mf are *italicized*.

**Figure 2:**
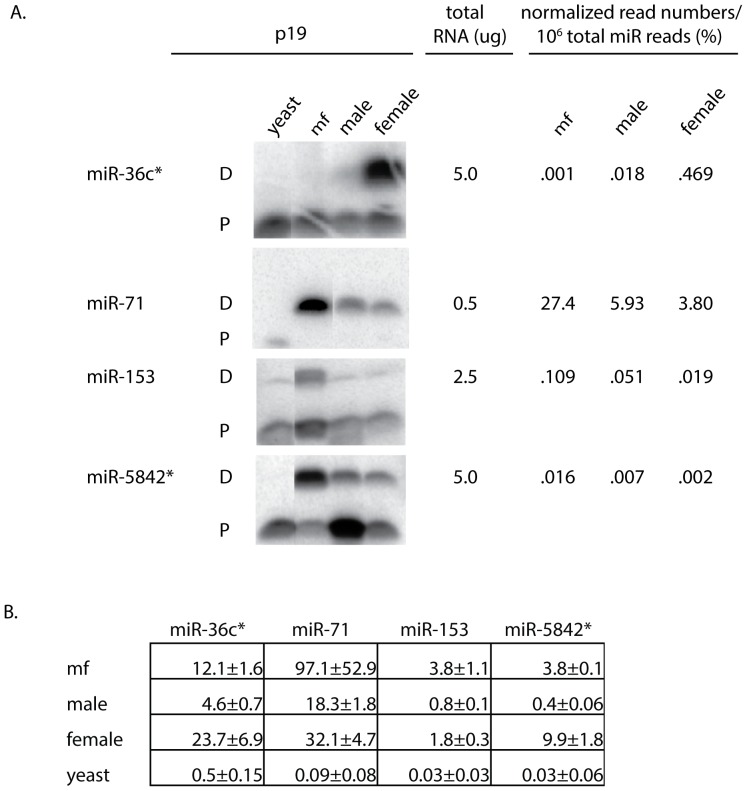
Quantitative detection of miRNAs by p19 and qPCR. (A) Total RNA from *B. malayi* mf, males, females and yeast (0.5–5.0 µg) was probed with a ^32^P-labeled miRNA specific ribo oligonucleotide followed by p19 detection. Eluted dsRNA duplexes were electrophoresed through 20% non-denaturing TBE gels. The positions of the double-stranded duplex (ds) and single-stranded oligonucleotide probe (ss) on each gel are denoted. Single-stranded probe can elute from the porous chitinase beads but represents <1% of the total probe added to p19 reactions [43]. The normalized read numbers of each miRNA in the mf, male and female CIP libraries (Table S2) are listed for comparison with the p19 results. (B) miRNA amounts were determined by qPCR for *B. malayi* mf, male and female RNA samples. The assays were performed in triplicate and the results are expressed as pg miRNA/µg of total RNA +/− one standard deviation.

Standard curves were generated using synthetic miR-71 RNA oligos to obtain a quantitative measure of this miRNA in different stages (Figures 2 and S1). Using p19, the amount of miR-71 in mf, females and males was calculated to be 178, 47 and 38 pg/µg of total RNA respectively. Using qPCR, the amounts of miR-71 in mf, females and males was calculated to be 97, 32 and 18 pg/µg of total RNA respectively. This is in agreement with the miR-71 sequencing read data which shows that mf have 3–5X more miR-71 then *B. malayi* adults (Figures 2, S1 and Table 4).

The autoradiographs of the p19 isolated miRNA:RNA probe duplexes show that miR-36c* is preferentially detected in RNA from female parasites, while miR-71, miR-153 and miR-5842* are more abundant in RNA from mf then adults (Figure 2). This is in agreement with the normalized read numbers from the CIP libraries (Figure 2 and Table S2). With the exception of miR-5842*, the qPCR data exhibited a similar pattern (Figure 2). Using qPCR, the quantity of miR-5842* was calculated to be 9.9 pg/µg in adult female RNA verses 3.8 pg/µg in mf RNA (Figure 2), a 2.6X difference. Additional validation using both qPCR and p19 detection methods at the same time and with the same RNA sample is needed to resolve this discrepancy.

### MicroRNA Conservation in the Hosts of Filarial Parasites

The Venn diagram in Figure 1 shows how the 99 *B. malayi* miRNA families are conserved in arthropods and vertebrates, the hosts of filarial parasites. When used to search miRBase (release 15), 61 (2/3) of the *B. malayi* miRNA families were found to be conserved in a wide range of organisms including arthropods, vertebrates and other helminths (Figure 1). Homologues were identified in vertebrates but not arthropods for 20 *B. malayi* miRNA families. For 7 of the 20 families, a homologue was identified in either *C. elegans* or *A. suum* [46], but for 13 *B. malayi* families, the only homologues identified were in *B. pahangi* or a vertebrate. For all except bma-miR-5844, homologues were identified in vertebrates infected by filarial parasites including humans, horses, cattle, pigs and platypus. In contrast, homologues were identified in arthropods but not vertebrates for 6 *B. malayi* miRNA families indicating that the miRNAs in these families may regulate targets specific to the ecdysozoa. Three of the 6 microRNAs (miR-45, -252 and -281*) have homologues in mosquitos, the invertebrate hosts of *Brugia*. Homologues could not be identified in either arthropods or vertebrates for 12 *B. malayi* miRNA families suggesting the possibility of helminth or even filarial-specific miRNA families.

### Putative filarial-specific miRNAs

Nine of the 12 *B. malayi* miRNA families identified only in helminths, appear to be specific to filarial parasites (Figure 1). Orthologues were identified for 8 of these in at least one other filarial species but not in other worms, arthropods or vertebrates (Table 5). No orthologues were identified for miR-9535 raising the possibility that this miRNA may be specific for *B. malayi*.

**Table 5:** Putative Filarial-Specific miRNAs.

miRNA	Wba^1^	Llo^1^	Ovo^1^	Bpa^2^	Sequence^3^	Acc. no.^2^^,^ 4	Position^2^^,^ 4	Strand	ΔG^2^^,^ 5
miR-2h*^6^	✓				AGUCGUAUCGGCUCUGAUAU	ADBV01012581	219–320	+	−28.6
miR-5365b	✓				UGAUUAAGAGAACACAAUCGA	ADBV01001310	7148–7251	+	−36.7
		✓			UGAUUAAGAGAACACAAUCGA	ADBU01002771	1844–1945	+	−36.6
			✓		UGAUUAAGAGAACACAAUCGA	ADBW01022104	310–411	−	−37.3
				✓	UGAUUAAGAGAACACAAUCGAAU	DS239359.1	262103–262022	−	ND
miR-5838*^6^	✓				UGCUAAACCGUAAAUGCUCCUA	ADBV01002682	1–79	−	ND
				✓	UGCUAAACCGUAAAUGCUCCUA	DS236978.1	839–732	−	ND
miR-5839				✓	AGGAGUAAUAUUCUAACGUUGAGCA	DS237141.1	12185–12289	+	ND
miR-5866	✓				UUACCAUGUUGAUCGAUCUCCA	ADBV01002064	4317–4416	−	−33.4
		✓			UUACCAUGUUGAUCGAUCUCCA	ADBU01003786	2402–2492	+	−30.4
				✓	UUACCAUGUUGAUCGAUCUCCA	DS239393.1	593278–593382	+	ND
miR-9534	✓				AUGUUAUUUUUUGAGGGAGUCGU	ADBV01011440	950–1060	+	−30.2
miR-9536	✓				CAUUCCAGGAAAGGCAUUGGAUA	ADBV01001305	5448–5547	+	−35
miR-9537	✓				UUUUCCUGCCUUCAUUUCUCUU	ADBV01003676	2186–2290	−	−39.6

1 The WGS sequence of *O.volvulus* (Ovo), *W. bancrofti* (Wba) and *L. loa* (Llo) were searched for orthologues using *B. malayi* miRNA hairpin sequences.

2 The *B. pahangi* data is from Winter *et. al.* [33].

3 Nucleotides that differ from the *B. malayi* miRNA sequence are underlined.

4 The accession number & nucleotide position defining a hairpin for each miRNA is shown.

5 ND =  not determined. The ΔG could not be determined for wba-miR-5838* because only a partial stemloop sequence is available.

6 When 2 miRNAs are excised from opposite arms of the same hairpin, a star (*) after the name generally denotes the less frequent form [25], [50].

**Figure 3:**
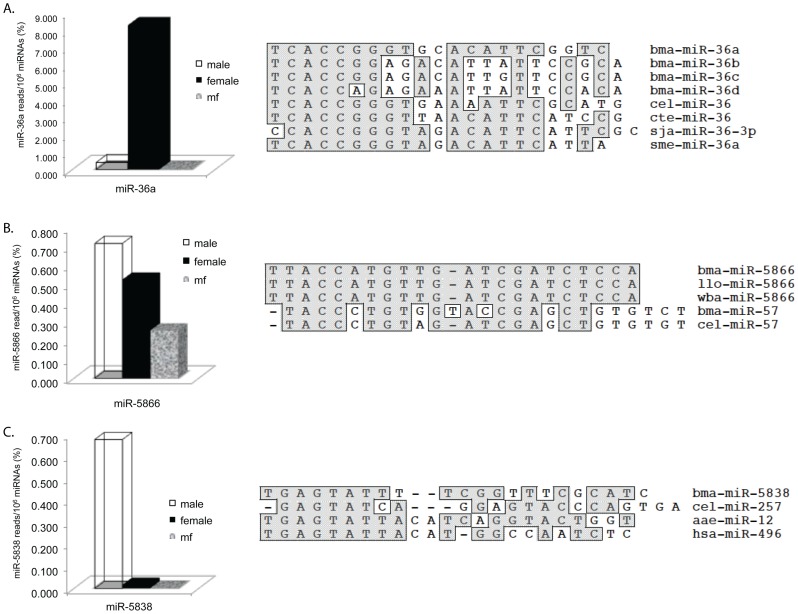
Alignment and Expression Analysis of *B. malayi* miRNAs 36a, 5866 and 5838. In each of the three panels (A: 36a, B: 5866, C: 5838), a bar graph depicting expression levels in the adult male, female and mf CIP libraries as well a ClustalW alignment of the miRNA with homologues is shown. Species abbreviations are as follows: bma, *B. malayi*; cel, *C. elegans*; cte, *Capitella teleta*; sja, *Schistosoma japonicum*; sme, *Schmidtea mediterranea*; llo, *L. loa*; wba, *W. bancrofti*; aae, *A. aegypti* and hsa, *Homo sapiens*.

Bma-miR-5866 is one of the most abundant filarial-specific miRNAs. It is found in all the libraries but is most prevalent in the male library where it represents ∼0.7% of the total miRNAs (Figure 3B and Table S2). Orthologues of bma-miR-5866 were identified in *B. pahangi* [33] as well as in the WGS data of *W. bancrofti* and *L. loa* [10]. The mature miRNA sequence is identical in the filarial species (Figure 3B and Table 5). Mature miR-5866 appears to be closely related to the miR-57 family. The miR-5866 seed sequence (TACCAT) is similar to the nucleotide sequence (TACCCT) at the 5′ end of *B. malayi* and *C. elegans* miR-57 (Figure 3B). Based on ClustalW alignments, bma-miR-5866 and bma-miR-57 are 67% identical overall. However, the filarial miR-5866 hairpin sequences only exhibit 34–40% identity with the hairpin sequences of *B. malayi* and *C. elegans* miR-57 (data not shown) suggesting that the miR-5866 and -57 families may have evolved from a common ancestor by gene duplication.

Of the putative filarial specific microRNAs, miR-9536 (Table S4) is one of the most interesting because of its location in an intron of Bm1_03065, a *cut-1* cuticlin gene fragment (genbank acc.#: XM_001892072). Cuticlin is the highly cross-linked and insoluble protein component of the nematode cuticle. Expression of the *C. elegans* cuticlin genes *cut-1*, *-3* and *-5* are tightly regulated at the transcriptional level and are involved with the formation of alae and control body shape [47]. Because of its location within an intron of Bm1_03065, miR-9536 expression is likely dependent on and coordinated with the expression of this cuticlin gene and suggests that its targets may be components of cuticlin biosynthesis or more generally involved with molting and cuticle synthesis.

These putative filarial specific miRNAs may be regulating some of the 20% of the proteome predicted to be the specific for *B. malayi* [9] such as pathways required for interactions with its vertebrate and arthropod hosts or with its *Wolbachia* endosymbiont [48].

### MicroRNA clusters

**Figure 4:**
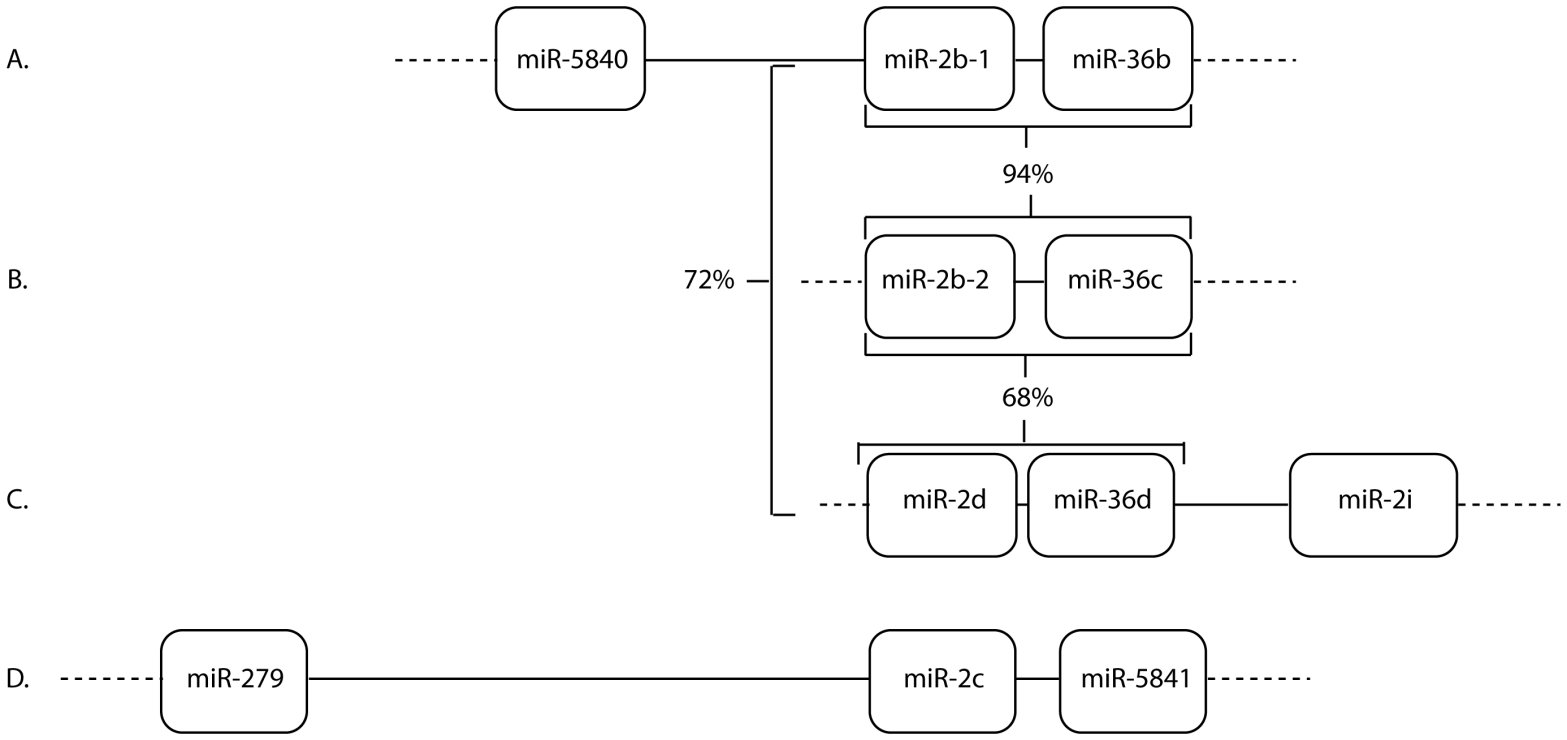
MicroRNA-2 clusters in *B. malayi*. The figure depicts the clusters in which a miR-2 paralogue is found within 2 kb of another miRNA (A–D). In clusters A–C, a miR-2 paralogue is clustered with a miR-36 paralogue. The % nucleotide identity shared between the three miR-2 -36 clusters is shown. Nucleotide identity was determined by aligning the region of each scaffold spanning a cluster (A: nt 1770–1984, B: nt 2496–2716 and C: nt 1716−1512) using Clustal W. The Genbank accession no. for each scaffold is A: DS237458, B: DS239143, C: DS238497 and D: DS237916.

In *B. malayi*, eight clusters containing a total of 19 miRNAs have been identified that span ≤2 kbps (Table S10) whereas in *C. elegans*, 18 clusters containing a total of 47 miRNAs can be found spanning ≤2 kb [49], [50]. Fewer miRNA clusters have been identified in *B. malayi* compared to *C. elegans*; however, new clusters may be identified upon completion of the *Brugia* genome. The majority of *C. elegans* clusters consist of multiple paralogues in a miRNA family. For example, 7 of the 8 miR-36 family members are clustered within 800 nt on chromosome II [51], [52]. In *B. malayi*, the only family members found clustered are miR-100a and -100d [32] as well as miR-2i and -2d (Figure 4 and Table S10).

### The *B. malayi* miR-2 family and clustering

**Figure 5:**
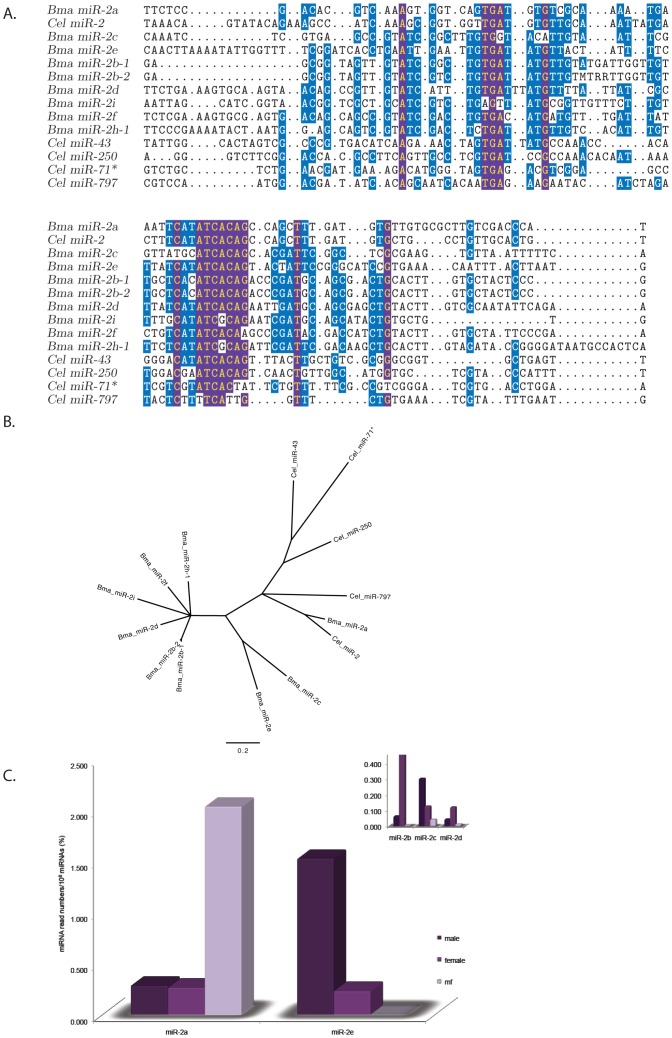
Alignment and Expression Analysis of the *B. malayi* miR-2 family. (A) Hairpin alignment of the miR-2 paralogues from *B. malayi* and *C.elegans*. Nucleotides conserved in ≥80% of the sequences making up the alignment are highlighted yellow in purple boxes. Nucleotides conserved in 50% of the sequences making up the alignment are highlighted white in blue boxes. The Bma-miR-2g hairpin is not included in the alignment because the mature miR is located on the 5′ arm not the 3′ arm, the location of the other mature miR-2 paralogues. (B). An un-rooted tree calculated from the alignment in A. The 0.2 bar represents nucleotide substitutions per site. (C). Bar graph depicting the expression levels of the prevalent *B. malayi* miR-2 paralogues (Tables 4, S2 and S3) in the adult male, female and mf CIP libraries. Bma-miR-2b, -2c and -2d are shown in the inset.

With 11 members (9 individual sequences +2 duplicates), the miR-2 family is the largest in *B. malayi* and twice the size of the 5 member *C. elegans* miR-2 family (Figure 5, Tables S5 and S6). The 6 nt consensus seed sequence, ATCACA is present in all family members except miR-2i and miR-2h which both have ATCGCA as a seed sequence (Figure 5A). ClustalW alignment of mature miR-2i and miR-2h exhibit 82 and 68% nt identity with miR-2d respectively, despite the single nt change in the seed sequence (data not shown). In addition, alignment of the *B. malayi* miR-2 hairpins demonstrates that a majority of the highly conserved nt blocks (highlighted purple in Figure 5A) are conserved in both the miR-2i (5/7) and miR-2h (6/7) hairpins.

The phylogenetic tree derived from the miR-2 hairpin alignment suggests that miR-2b, -2d, -2f, -2i and -2h are derived from a common ancestor that underwent an expansion event (Figure 5B). It also demonstrates that bma-miR-2a and cel-miR-2 are the most closely related family members being 100% identical [32].

Bma-miR-2a, -2b and -2e are the most highly expressed of the *B. malayi* miR-2 paralogues but have different patterns of expression (Figure 5C, Tables 4 and S2). Bma-miR-2a represents ∼2% of the miRNA population in mf, about 7.5X higher than the levels of this miRNA in either the male or female libraries whereas bma-miR-2b, -2c, -2d and -2e are all expressed in the adult libraries, none of them are found to be expressed at significant levels in the mf library (Figure 5C, Tables 4 and S2). The remaining *B. malayi* miR-2 paralogues (miR-2f, -2g, -2h and -2i) are only present at very low levels (<100 reads) in the libraries (Table S4).

About half of the *B. malayi* miR-2 family members are located in clusters; most with paralogues of the miR-36 family (Figure 4 and Table S10). The miR-2b, -36 clusters represent a possible duplication event as they are 94% identical (Figure 4, scaffolds A and B). The miR-2d, -36d cluster on scaffold C may also represent a duplication event as it is 72 and 68% identical to the miR-2b, -36 clusters on scaffolds A and B respectively. A fourth miR-2 paralogue, miR-2i, is also found clustered on scaffold C with miR-2d and -36d (Figure 4 and Table S10). Using an updated assembly of the *B. malayi* genome, miR-2 clusters A-C were found arranged in 2 groups along the length of a 23 kb scaffold with a fourth miR-2 cluster consisting of miR-2h-2, miR-5839 and miR-2f (data not shown, personal communication, Elodie Ghedin).

Unlike *B. malayi*, *C. elegans* miR-2 family members are not clustered with one another but with paralogues of the miR-44 family. Only one *C. elegans* miR-2 paralogue, miR-43, is found clustered with a miR-36 paralogue, miR-42 [49].

Clustering may result in coordinated expression. For example, miR-2b and its cluster partners miR-36c and -36b are more highly expressed in adult parasites, particularly females compared to mf (Tables 4 and S2) confirming previous Northern blot results [32]. In addition, the expression patterns of miR-2d and -36d as well as miR-2c and -5841 are also enhanced in adults compared to mf (Tables 4 and S2). Additional studies are necessary to determine if these clustered miRNAs are processed from the same RNA transcripts.

Although, the exact function of the miR-2, -36 clusters in *B. malayi* must await target identification, recent work in other systems suggests that they have a role in regulating programmed cell death. In *D. melanogaster, the* miR-2 family suppresses apotosis during embryogenesis by targeting the 3′ UTRs of pro-apototic genes [53]. In addition, RISC immunoprecipitation studies identified the BH3-like pro-apototic gene, *egl-1*, [54], as a target of the miR-36 family in embryos [55].

### Stage enhanced expression of miRNAs in *B. malayi*

Deep sequencing is very useful in determining relative expression levels of the same miRNA because it avoids the bias observed when comparing different sequences [56], [57]. The number of normalized reads can be used for quantitative comparisons of miRNA levels in different developmental stages. To compare miRNAs levels across libraries, the raw read numbers of each miRNA in a library was normalized to 1 million total miRNA reads (Tables S1 and S2). Using this methodology, about 2/3 (51/85) of the prevalent miRNAs, excluding duplicates (Table S3), are enriched in one CIP library over another as defined by a 5X difference in the % of normalized reads/million miRNA reads total (Tables 4 and S2).

Strikingly, 31 miRNAs are at least 5X more highly expressed in adults than mf while only 4 miRNAs (miR-2a, -71, -92 and -5366*) are more highly expressed in mf (Tables 4 and S2).

For example, miR-71 represents ∼27% of the total miRNAs in mf and is 4.6 and 7.2X more abundant than the levels of miR-71 observed in either the male or female CIP libraries, respectively (Tables 4 and S2). The levels of miR-71 in the CIP libraries were also validated using p19 and qPCR, (see section titled, “MicroRNA validation using p19 and qPCR”). In *C. elegans*, miR-71 promotes longevity [58], [59]. Knockouts of miR-71 shortened the lifespan of mutant worms from about 20 to 10 days and this effect is mediated through activation of the DAF-16/FOXO transcription factor in the intestines [59], [60]. In addition, miR-71 levels are up-regulated in L1 diapause and dauer larvae [61].

Our finding that miR-71 is one of the most abundantly expressed miRNAs in *B. malayi* mf suggests that it may function to regulate the longevity of mf which can circulate throughout the body for up to a year waiting to be ingested by a mosquito [3], [62]. Homologues of several of the proteins predicted to be targeted by miR-71 in *C.elegans* have been identified in *B. malayi* including *pdk-1* (genbank acc. EDP33519) and *Daf-16* (genbank acc. XP_001901487).

Of the 31 miRNAs expressed more highly in adults than mf, 11 miRNAs are more highly expressed in females than males; 11 miRNAs are more highly expressed in males while 9 miRNAs appear to be expressed at about the same levels in both sexes (Tables 4 and S2).

For example, miR-36a one of the 4 miR-36 paralogues identified in *B. malayi*, represents 8% of the total miRNA population in the female CIP library but only ∼0.4% of miRNAs in the male library (a 20X difference) and is only found at trace levels in mf. *B. malayi* miR-36c and -36b are also predominately expressed in the female CIP library but at lower levels compared to miR-36a (Figure 3A, Tables 4 and S2). Based on Clustal W alignment, bma-miR-36a is closely related to (76% nt identity) miR-36 homologues from a wide variety of helminths including *C. elegans*, the marine polychaete *Capitella teleta*, the flatworm *Schmidtea mediterranea* and the parasitic blood fluke *Schistosoma japonicum* (Figure 3A). Unlike the other *B. malayi* miR-36 paralogues, miR-36a is not clustered with a miR-2 paralogue (Figure 4).

In *C.elegans*, the miRNA-35 family (miRNAs 35–42) is required for embryonic development. Its deletion results in embryonic and L1 lethality [51]. Interestingly, most expression of this family originates in the gonad during oogenesis and to a lesser extent during spermatogenesis [52].

Bma-miR-5838 is one of the 11 miRNAs more abundant in the male than the female CIP library. It represents ∼0.7% of miRNAs in the male CIP library compared to only .02% in the female library (a 35X difference; Figure 3C, Tables 4 and S2). Clustal W alignment shows that bma-miR-5838 shares 8 nt at its 5′ end which includes the seed sequence, GAGTAT, with *Aedes aegypti* miR-12 and human miR-496 (Figure 3C).

Future work will involve sequencing small RNA libraries from germline tissue dissected from adult parasites to determine whether the enhanced expression of miRNAs in adults derives from somatic or germline origins.

### MicroRNA Target Identification and Chemotheraputic Development

The developmental stages of filarial parasites that transition between insect and vertebrate hosts have traditionally been targeted for chemotherapeutic control. However, the high expression levels of some miRNAs in adult parasites (Table 4) suggests that they might be useful for the design of macrofilaricides.

Although miRNAs target hundreds of transcripts, recent work indicates that miRNAs often target functionally related mRNAs within a biochemical pathway [59], [61], [63] and are often in feedback loops with transcription factors which themselves may regulate transcription of functionally related genes [64], [65]. For example, miR-71 was found to target multiple components of the insulin-like pathway in *C. elegans* including the forkhead box O transcription factor, *Daf-16* [59], [60]. An association between miRNAs and transcription factors likely occurs in *B. malayi* as well. Bma-miR-5855 is located within an intron of Bm1_31015, annotated as a putative transcription factor and homologue of *C. elegans* unc-3 (Table S4).

The development of chemotherapeutics against filarial-specific miRNAs provides a novel way to distrupt post-transcriptional regulation in parasites without disturbing the host's miRNA regulatory networks. Filarial–specific miRNAs might be targeted directly using modified antisense RNAs such as antagomirs [66], [67]. Recent developments in antagomir technology have demonstrated that intravenously injected 8 mer locked nucleic acid oligonucleotides are readily distributed throughout mouse tissue and can efficiently silence whole miRNA families (the same seed sequence) with negligible off-target effects [68].

Using miRDeep [42], 145 miRNAs were predicted from ∼30 million small RNA sequencing reads cloned from *B. malayi* adults and mf. The miRNAs segregate into 99 families each defined by a unique seed sequence. Two ligase independent methods, quanitative PCR and p19 [43] used to determine the quantity of four miRNAs from different stages of *B. malayi* showed good correlations with the sequencing read numbers from the libraries. Comparisons of the miRNA expression levels from the three CIP libraries demonstrated that about a third of the *B. malayi* miRNAs are differentially expressed. Sixty-one of the *B. malayi* families are widely conserved in arthropods, vertebrates and helminths. Approximately half of the families have a homologue with a seed match in *B. pahangi* and *C. elegans*. The miR-2 family, the largest in *B. malayi* with 11 paralogues, has expanded compared to *C. elegans* with 5 paralogues, respectively. Interestingly, homologues of 20 *B. malayi* miRNA families are conserved in vertebrates but not in other helminths or arthropods. Nine miRNA families appear to be filarial-specific as homologues were not identified in other organisms.

Characterization of the miRNAs from *B. malayi* is an important first step in determining the miRNA regulatory network in filarial parasites. Unlike free-living nematodes, filarial parasites encounter pronounced environmental changes when they transition between vertebrate and insect hosts. In addition, they must contend with their host's immune responses. It is likely that miRNAs have developed critical roles in these processes that could be considered for anti-filaricidals. Future work to identify the targets of these miRNAs, particularly of the filarial-specific miRNAs, may lead to new approaches for the development of therapies against this debilitating disease.

## Materials and Methods

### RNA isolation

RNA was isolated from *B. malayi* males, females and mf (TRS Laboratories, Athens, GA) using Trizol (Invitrogen) with a steel ball bearing as described previously [32].

### Small RNA cloning and nucleotide sequencing

Small RNAs (18 to 30 nt) from *B. malayi* males, females and microfilariae were gel purified and cloned using a 5′ ligation - dependent protocol [36]. Essentially, the 5′ ends of the small RNAs were dephosphorylated with calf intestinal phosphatase (CIP) from New England Biolabs before ligation of their 3′ ends to a preadenylated DNA oligo. The 3′-ligated-RNAs were rephosphorylated with polynucleotide kinase (PNK) and ligated to a 5′ linker prior to first strand cDNA synthesis and amplification with Solexa sequencing primers. In addition, a direct (DIR) and a Tobacco Acid Pyrophosphatase (TAP) small RNA library were also prepared from mf. In the direct library, small RNAs were ligated to the 3′ preadenylated DNA oligo and 5′ linker without pretreatment with CIP and polynucleotide kinase. In the TAP library, small RNAs were incubated with Tobacco Acid Pyrophosphatase (Epicentre) that digests capped and multi-phosphorylated small RNA ends to monophosphates prior to ligation with the preadenylated DNA oligo and 5′ linker [36]. The cDNA libraries were sequenced by the University of Massachusetts (Worcester, MA) Deep Sequencing Core using an Illumina Genome Analyzer II.

### Sequencing data analysis

#### Small RNA library profiles

As a basis for comparing the sequenced libraries, a profile was generated for each library that consists of four descriptive metrics. The first is the total number of raw reads returned from the Illumina sequencer. After removing the 3′ linker (identified by its first 6 nt, CTGTAG) from the raw reads, all inserts <17 nts long are discarded. The number of remaining reads with inserts ≥17 nt long comprise the second metric of the profile. This set of reads was then aligned to the *B. malayi* genome (Genbank accession #s: DS236884 to DS264093) and to the sequence of the 18S rRNA gene (GI: 2707744) using RazerS [69] with the alignment parameters set at 100% recognition rate and 100% identity. All reads that aligned to more than 100 locations were discarded to avoid reads that match to low complexity regions of the genome. The number of reads that are an exact match to the *B. malayi* genome and the number of 18S rRNA matching reads comprise the third and fourth metric of the profile respectively. The processed reads from each of the five sequenced libraries are in Files S1–S5.

#### miRDeep Analysis

MicroRNAs were primarily identified in the filtered Illumina sequencing data (inserts ≥17 nt long after removal of the 3′ linker) using miRDeep with default settings [42]. The miRDeep algorithm is based on the model of miRNA biogenesis [70]. MiRDeep maps sequencing reads onto a genomic sequence then extracts the DNA sequence bracketing these alignments and determines whether the DNA sequence folds into a hairpin consistent with miRNA biogenesis. The miRDeep output was manually curated to confirm miRNA identifications.

Because the *B. malayi* genome is only 75–80% complete [9], the filtered illumina sequencing data was also searched directly with the full length sequences of highly conserved miRNAs or with conserved 7 nt seed sequences to identify miRNAs missed by miRDeep.

#### Calculation of miRNA prevalence in the CIP RNA libraries

The filtered libraries containing only inserts ≥17 nucleotides long (see small RNA library profiles) were searched for the mature form of each miRNA as well as for shorter and longer forms (to a maximum of ±4 nucleotides). For each library, the total number of reads of each form of a miRNA was reported as well as the sum of all the forms. In order to compare miRNA levels across CIP libraries, the number of reads of each miRNA was normalized to 1 million total miRNA reads. A 5X difference in the expression level of a miRNA between normalized samples was chosen as the minimum threshold for differential expression.

### MicroRNA family assignments

To identify paralogues, *B. malayi* miRNAs were aligned using BioPython's [71] pairwise2 module using match/mismatch scores of 1, −3 and gap open/extend penalties of −5/−2. MicroRNAs were grouped into families if they shared a minimum of 6 consecutive nt with no mismatches in the first 9 bases of each sequence or if the global alignment between miRNAs exhibited a minimum of 60% identity [40]. A more stringent refinement of the initial output was made before making final family assignments using the following criteria: miRNAs were grouped into families if they shared a minimum of 6 contiguous nt in common between positions 1–6 or 2–7 [16]. Without nt identity at the 5′ end, miRNAs were only considered family members if they exhibited ≥70% nucleotide identity overall [40]. Paralogous miRNA families in *C. elegans* (miRBase release 15, http://www.mirbase.org/) and *B. pahangi* [33] were identified using this same protocol. To identify homologues in other species, *B. malayi* miRNAs were aligned with *C. elegans*, human and mosquito (*Aedes aegypti*, *Anopheles gambiae* and *Culex quinquefasciatus*) miRNAs downloaded from miRBase (release 15) and *B. pahangi* miRNAs [33] using the above protocol. When a homologue was not identified, miRBase was searched directly for a seed match to any arthropod or vertebrate miRNA.

### Bioinformatic Identification of miRNAs in Related Filarids

MicroRNAs were identified in related filariads as described by [32] except that *B. malayi* miRNA hairpin sequences were used to search the whole genome shotgun (WGS) assemblies of *Wuchereria bancrofti* (Genbank acc. ADBV00000000), *Onchocerca volvulus* (Genbank acc. ADBW00000000) and *Loa loa* (Genbank acc. ADBU0000000) at NCBI (http://www.ncbi.nlm.nih.gov/) with BLASTN (default settings).

### Phylogenetic Analysis

Hairpin sequences were aligned using MAFFT v6.833b [72] run with RNA structural alignment options: mafft-xinsi –reorder –kimura 1 –ep 0.0. The bma-miR-2g hairpin was excluded from the alignment because the mature miRNA is on a different arm of the hairpin compared to all the other sequences. A Baysian Inference tree was calculated from the aligned sequences using MrBayes [73] at Phylogeny.fr (http://www.phylogeny.fr/) [74]. MrBayes v3.1.2 was run for 100000 generations with trees sampled every 10 generations. The likelihood model used 6 substitution types, invariable+gamma rate variation across sites, and the default (4by4) substitution model. The first 2500 trees were discarded when calculating the consensus to allow the models to converge.

### MicroRNA detection with the p19 dsRNA binding protein

*B. malayi* miR-71, miR-36c*, mir-153 and miR-5842* identified in the small RNA libraries were confirmed using the p19 miRNA detection kit (NEB, [43]). Essentially, total RNA (0.5–5.0 µgm) from *B. malayi* mf, males and females was mixed with 1–2 ng of a ^32^P-labeled probe in 1X p19 binding buffer and hybridized for 2 hrs at 65°C with the exception of the miR-71 samples which were hybridized at 55°C. Typically, 50 ng of probe was end-labeled with [γ - ^32^P] ATP (10 mCi/ml, 6000 Ci/mmol; Perkin Elmer) using T4 polynucleotide kinase (NEB). After heat killing the kinase, unincorporated label was separated from the probe by passing the reaction over an illustra microspin G-25 column (GE Healthcare). The RNA oligonucleotide probes (Integrated DNA Technologies) used for miRNA enrichment are as follows:

miR-36c*: 5′ ACCGUGAGAGACUAUCCCG 3′;

miR-71: 5′ UCACUACCCAUGUCUUUCA 3′;

miR-153: 5′ CACUUUUGUGACUAUGCAA 3′ and

miR-5842*: 5′ UAGCAGGAUGUAUCCAUCG 3′.

Following hybridization, p19 beads (10 µl) were added and the reactions were incubated at RT for 2 hrs with shaking. After washing the p19 beads and elution, the miRNA: ^32^P-labeled probe duplexes were electrophoresed through 20% acrylamide, non-denaturing TBE gels (Invitrogen) along side controls. Tortula yeast RNA (0.5–5.0 µg; US Biological) was used as a negative control. For positive controls, yeast RNA was spiked with 1–2 ng of a miRNA oligonucleotide (Table S3; Integrated DNA Technologies) complementary to the probe. Control samples were processed along side the *B. malayi* RNA samples.

After electrophoresis, the gels were dried and exposed to a Storage Phosphor Screen GP (Kodak) for 1–6 days that was then scanned using a Typhoon 9400 variable mode imager (GE Healthcare).

#### MicroRNA detection using qPCR

qPCR was performed essentially as described [75] with the following modifications: For cDNA synthesis, 1 µg of total *B. malayi* RNA from either mf, males or females was added to a 20 µl reaction containing 200 units of M-MuLV reverse transcriptase (NEB), 2 units of *E. coli* poly (A) polymerase (NEB), 10 µM tagged oligo d(T)23VN primer (5′ GGAGACAUGGATCCCCATGGAA(T)_23_VN 3′) in 1X PAP/M-MuLV buffer (50 mM TrisHCl pH 8.1, 0.1167 mM NaCl, 8.0 mM MgCl_2_, 25 mM KCl, 3.33 mM DTT, 0.1 mM ATP and 1 mM each dNTP). The reaction was incubated for 30 min at 37°C then heat inactivated at 65°C for 20 min. Samples were diluted to a final volume of 100 µl with water for use in qPCR.

For qPCR, 1 µl of cDNA was mixed with 0.5 µM of a microRNA specific primer, 10 µM extension primer (5′ GGAGACAUGGATCCCCATGGAA 3′), 12.5 µl IQ SYBR Green Supermix 2x (Biorad) in a final volume of 25 µl. Tortula yeast and *E. coli* RNA were used as negative controls. A cDNA synthesis reaction using synthetic miR-71 was used to generate a standard curve. The reactions were heated at 95°C for 5 min and then cycled 40 times at 95°C for 15 sec, 50°C for 15 sec, and 72°C for 30 sec in a BioRad C1000 thermal cycler with a CFX96 real time system. The four microRNAs were amplified in triplicate from each of the three *B. malayi* cDNAs. Values are reported as pg miRNA/µg of total RNA plus/minus standard deviation. The microRNA specific primers used for qPCR are as follows: miR36c* (5′ CGG GAU AGU CUC UCA CGG UAG AGC 3′), miR-71 (5′ UGA AAG ACA UGG GUA GUG AGA 3′), miR-153 (5′ UUG CAU AGU CAC AAA AGU GAU GG 3′) and miR-5842* (5′ CGA UGG AUA CAU CCU GCU AGU U 3′).

## Supporting Information

Figure S1 **Quantitation of miR-71 in** ***B. malayi*** **using p19.** A standard curve was generated by hybridizing defined quantities of synthetic *B. malayi* miR-71 with 1 ng of ^32^P-labeled miR-71 probe as described [43]. (A) Autoradiograph of a 20% non-denaturing gel showing the miR-71:probe hybrid eluted from p19 beads. The mobility of the double-stranded (ds) miRNA:RNA probe duplex and single-stranded (ss) RNA probe is shown to the right of the gel. (B) The amount of radioactivity in 10 µl (1/5^th^) of the eluted miR-71: probe hybrid was measured by scintillation counting and plotted against known amounts of synthetic miR-71 to generate a standard curve. (C) Autoradiograph of a non-denaturing gel showing p19 eluants of miR-71 from RNA samples of *B. malayi* males (M), females (F), microfilariae (mf) and third stage larvae (L3). The position of the double-stranded (ds) miRNA:RNA probe duplex and single-stranded (ss) RNA probe is shown to the right of the gel. (D) The quantity of miR-71 in the different stages of *B. malayi* was calculated from the standard curve using the amount of radioactivity in 10 µl of p19 eluant.
(TIFF)

Table S1 *B. malayi* miRNA read numbers. The total number of reads for each miRNA from the five different libraries are listed in the table. The three CIP libraries (male, female and mf) were generated by treatment of small RNAs with calf intestinal phosphatase followed T4 polynucleotide kinase. This allows ligation and sequencing of all RNAs regardless of phosphorylation status. Two additional microfilarial RNA libraries were also prepared. The mf DIR library was made by direct ligation of linkers without phosphatase treatment. The DIR protocol enables ligation and sequencing of small RNAs processed by Dicer that already have a 5′ phosphate. The mf TAP library was prepared using tobacco acid pyrophosphatase to convert capped and multiphosphorylated small RNA ends to monophosphates prior to ligation. The miRNAs were identified using miRDeep from filtered sequence reads as described in Materials and Methods. The number of reads is not corrected for the size difference of the libraries. The miRNAs identified in the DIR and/or TAP but not the CIP libraries are boxed in this table.
(XLSX)

Table S2 Prevalence of *B. malayi* miRNAs in the small RNA libraries. The raw reads numbers (Table S1) for each miRNA were normalized to a theoretical 10^6^ total miR reads and are presented as a % of the total miRNA reads in a given library. Values highlighted red indicate a 5X increase in the relative number of reads in one CIP library over another.
(XLSX)

Table S3 Prevalent *B. malayi* miRNAs. This table lists the sequence, accession number, position, strand, hairpin stability and method(s) of identification/validation for the prevalent *B. malayi* miRNAs: those represented by 100 or more reads in one of the small RNA libraries (Table S1). Rare miRNAs are listed in Table S4. When two miRNAs are generated from the same RNA hairpin, a star (*) after the name denotes the less abundant miRNA.
(XLSX)

Table S4 Rare *B. malayi* miRNAs. This table lists the sequence, accession number, position, strand, hairpin stability and method(s) of identification/validation for rare *B. malayi* miRNAs: those represented by fewer than 100 reads in the raw data (Table S1). Prevalent miRNAs are listed in Table S3. When two miRNAs are generated from the same RNA hairpin, a star (*) after the name denotes the less abundant miRNA.
(XLSX)

Table S5 *B. malayi* miRNA paralogues. For each miRNA family, the table lists *B. malayi* miRNAs that are related by either a 5′ 7 nt match at positions 1–7 or 2–8, a 5′ 6 nt match at positions 1–6 or 2–7 or by overall identity of ≥70% with no 5′ match. In addition, miRNAs with a 5′ nt match that also exhibit overall identity of 60–69% are denoted by a ∧ symbol.
(XLSX)

Table S6 *C. elegans* miRNA paralogues. For each miRNA family, the table lists *C. elegans* miRNAs that are related by either a 5′ 7 nt match at positions 1–7 or 2–8, a 5′ 6 nt at positions 1–6 or 2–7 or by overall identity of ≥70% with no 5′ match. In addition, miRNAs with a 5′ nt match that also exhibit overall identity of 60–69% are denoted by a ∧ symbol.
(XLSX)

Table S7 Homologous miRNAs in *B. malayi* and *C. elegans*. For each miRNA family, the table lists the *B. malayi* members that have homologues in *C. elegans*. The relationship is indicated as either a 5′ 7 nt match at positions 1–7 or 2–8, a 5′ 6 nt at positions 1–6 or 2–7 or by overall identity of ≥70% with no 5′ match. In addition, those miRNAs with a 5′ nt match that also exhibit overall identity of 60–69% are denoted by a ∧ symbol.
(XLSX)

Table S8 *B. pahangi* miRNA paralogues. For each miRNA family, the table lists *B. pahangi* miRNAs that are related by either a 5′ 7 nt match at positions 1–7 or 2–8, a 5′ 6 nt at positions 1–6 or 2–7 or by overall identity of ≥70% with no 5′ match. In addition, miRNAs with a 5′ nt match that also exhibit overall identity of 60–69% are denoted by a ∧ symbol.
(XLSX)

Table S9 Homologous miRNAs in *B. malayi* and *B. pahangi*. For each miRNA family, the table lists the *B. malayi* members that have homologues in *B. pahangi*. The relationship is indicated as either a 5′ 7 nt match at positions 1–7 or 2–8, a 5′ 6 nt at positions 1–6 or 2–7 or by overall identity of ≥70% with no 5′ match. In addition, those miRNAs with a 5′ nt match that also exhibit overall identity of 60–69% are denoted by a ∧ symbol.
(XLSX)

Table S10 *B. malayi* miRNA clusters. The table lists the miRNAs that are clustered in the *B. malayi* genome. The orientation (forward or reverse strand) and relative position of the miRNAs in a cluster is based on the annotated *B. malayi* genome. The bp distance between the miRNAs in a cluster is shown in the right hand column. MicroRNA-9 and miR-79 are on opposite arms of the same hairpin.
(XLSX)

File S1 **Female CIP library.** Solexa reads from the female *B. malayi* small RNA library. The RNA for this library was treated with CIP and then T4 PNK prior to linker ligation allowing ligation of all small RNAs regardless of their phosphorylation status. These reads are ≥17 nt long and an exact match to the *B. malayi* genome. Each sequence in the library is preceded by a line containing the following information: 1) the library from which the sequence is derived (F =  female), 2) a unique alphanumeric identifier for that sequence and 3) the number of reads for that sequence in the library.
(ZIP)

File S2 **Male CIP library.** Solexa reads from the male *B. malayi* small RNA library. The RNA for this library was treated with CIP and then T4 PNK prior to linker ligation allowing ligation of all small RNAs regardless of their phosphorylation status. These reads are ≥17 nt long and an exact match to the *B. malayi* genome. Each sequence in the library is preceded by a line containing the following information: 1) the library from which the sequence is derived (M =  male), 2) a unique alphanumeric identifier for that sequence and 3) the number of reads for that sequence in the library.
(ZIP)

File S3 **Microfilarial CIP library.** Solexa reads from the *B. malayi* mf small RNA library. The RNA for this library was treated with CIP and then T4 PNK prior to linker ligation allowing ligation of all small RNAs regardless of their phosphorylation status. These reads are ≥17 nt long and an exact match to the *B. malayi* genome. Each sequence in the library is preceded by a line containing the following information: 1) the library from which the sequence is derived (U = mf), 2) a unique alphanumeric identifier for that sequence and 3) the number of reads for that sequence in the library.
(ZIP)

File S4 **Microfilarial TAP library.** Solexa reads from the *B. malayi* mf TAP small RNA library. The RNA for this library was treated with tobacco acid pyrophosphates, TAP, prior to linker ligation allowing ligation and sequencing of all small RNAs that may have a 5′ cap or 5′ triphosphate. These reads are ≥17 nt long and an exact match to the *B. malayi* genome. Each sequence in the library is preceded by a line containing the following information: 1) the library from which the sequence is derived (U_TAP = mf TAP), 2) a unique alphanumeric identifier for that sequence and 3) the number of reads for that sequence in the library.
(ZIP)

File S5 **Microfilarial DIR library.** Solexa reads from the *B. malayi* mf DIR small RNA library. The RNA for this library was not treated enzymatically prior to linker ligation. This selects for RNAs such as miRNAs, that already have a 5′ phosphate and a 3′ OH. These reads are ≥17 nt long and an exact match to the *B. malayi* genome. Each sequence in the library is preceded by a line containing the following information: 1) the library from which the sequence is derived (U_DIR = mf DIR), 2) a unique alphanumeric identifier for that sequence and 3) the number of reads for that sequence in the library.
(ZIP)

## References

[1] WHO (1992) Lymphatic filariasis: the disease and its control. Fifth report of the WHO Expert Committee on Filariasis. 0512-3054 (Print)0512-3054 (Linking). 1–71 p. 1441569

[2] Scott AL (2000) Lymphatic-dwelling Filariae. In: Nutman TB, editor. Lymphatic Filariasis. London: Imperial College Press. pp. 5–40.

[3] Simonsen PE (2009) Filariases. In: Cook G, Manson P, Zumla A, editors. Manson's Tropical Diseases. 22 ed: Saunders, Elsevier. pp. 1477–1513.

[4] Kumaraswami V (2000) The Clinical Manifestations of Lymphatic Filariasis. In: Nutman TB, editor. Lymphatic Filariasis. London: Imperial College Press. pp. 103–126.

[5] Molyneux DH, Taylor MJ (2001) Current status and future prospects of the Global Lymphatic Filariasis Programme. Curr Opin Infect Dis 14: 155–159. 11979126 10.1097/00001432-200104000-00008

[6] Addiss DG, Dreyer G (2000) Treatment of Lymphatic Filariasis. In: Nutman TB, editor. Lymphatic Filariasis. London: Imperial College Press. pp. 151–200.

[7] Bourguinat C, Ardelli BF, Pion SD, Kamgno J, Gardon J, et al (2008) P-glycoprotein-like protein, a possible genetic marker for ivermectin resistance selection in *Onchocerca volvulus* . Mol Biochem Parasitol 158: 101–111. 18215431 10.1016/j.molbiopara.2007.11.017

[8] Williams SA, Lizotte-Waniewski MR, Foster J, Guiliano D, Daub J, et al (2000) The filarial genome project: analysis of the nuclear, mitochondrial and endosymbiont genomes of *Brugia malayi* . Int J Parasitol 30: 411–419. 10731564 10.1016/s0020-7519(00)00014-x

[9] Ghedin E, Wang S, Spiro D, Caler E, Zhao Q, et al (2007) Draft genome of the filarial nematode parasite *Brugia malayi* . Science 317: 1756–1760. 17885136 10.1126/science.1145406 PMC2613796

[10] Desjardins CA, Cerqueira GC, Goldberg JM, Dunning Hotopp JC, Haas BJ, et al (2013) Genomics of Loa loa, a Wolbachia-free filarial parasite of humans. Nature genetics 45: 495–500. 23525074 10.1038/ng.2585 PMC4238225

[11] Godel C, Kumar S, Koutsovoulos G, Ludin P, Nilsson D, et al (2012) The genome of the heartworm, Dirofilaria immitis, reveals drug and vaccine targets. FASEB journal: official publication of the Federation of American Societies for Experimental Biology 26: 4650–4661. 22889830 10.1096/fj.12-205096 PMC3475251

[12] Lee RC, Feinbaum RL, Ambros V (1993) The *C. elegans* heterochronic gene *lin-4* encodes small RNAs with antisense complementarity to *lin-14* . Cell 75: 843–854. 8252621 10.1016/0092-8674(93)90529-y

[13] Wightman B, Ha I, Ruvkun G (1993) Posttranscriptional regulation of the heterochronic gene *lin-14* by *lin-4* mediates temporal pattern formation in *C. elegans* . Cell 75: 855–862. 8252622 10.1016/0092-8674(93)90530-4

[14] Bartel DP (2004) MicroRNAs: genomics, biogenesis, mechanism, and function. Cell 116: 281–297. 14744438 10.1016/s0092-8674(04)00045-5

[15] Bartel DP (2009) MicroRNAs: target recognition and regulatory functions. Cell 136: 215–233. 19167326 10.1016/j.cell.2009.01.002 PMC3794896

[16] Lewis BP, Burge CB, Bartel DP (2005) Conserved seed pairing, often flanked by adenosines, indicates that thousands of human genes are microRNA targets. Cell 120: 15–20. 15652477 10.1016/j.cell.2004.12.035

[17] Friedman RC, Farh KK, Burge CB, Bartel DP (2009) Most mammalian mRNAs are conserved targets of microRNAs. Genome research 19: 92–105. 18955434 10.1101/gr.082701.108 PMC2612969

[18] Grimson A, Farh KK, Johnston WK, Garrett-Engele P, Lim LP, et al (2007) MicroRNA targeting specificity in mammals: determinants beyond seed pairing. Molecular cell 27: 91–105. 17612493 10.1016/j.molcel.2007.06.017 PMC3800283

[19] Shin C, Nam JW, Farh KK, Chiang HR, Shkumatava A, et al (2010) Expanding the microRNA targeting code: functional sites with centered pairing. Molecular cell 38: 789–802. 20620952 10.1016/j.molcel.2010.06.005 PMC2942757

[20] Brennecke J, Stark A, Russell RB, Cohen SM (2005) Principles of microRNA-target recognition. PLoS biology 3: e85. 15723116 10.1371/journal.pbio.0030085 PMC1043860

[21] Behm-Ansmant I, Rehwinkel J, Doerks T, Stark A, Bork P, et al (2006) mRNA degradation by miRNAs and GW182 requires both CCR4:NOT deadenylase and DCP1:DCP2 decapping complexes. Genes Dev 20: 1885–1898. 16815998 10.1101/gad.1424106 PMC1522082

[22] Eulalio A, Huntzinger E, Izaurralde E (2008) GW182 interaction with Argonaute is essential for miRNA-mediated translational repression and mRNA decay. Nat Struct Mol Biol 15: 346–353. 18345015 10.1038/nsmb.1405

[23] Prochnik SE, Rokhsar DS, Aboobaker AA (2007) Evidence for a microRNA expansion in the bilaterian ancestor. Dev Genes Evol 217: 73–77. 17103184 10.1007/s00427-006-0116-1

[24] Wheeler BM, Heimberg AM, Moy VN, Sperling EA, Holstein TW, et al (2009) The deep evolution of metazoan microRNAs. Evol Dev 11: 50–68. 19196333 10.1111/j.1525-142X.2008.00302.x

[25] Lau NC, Lim LP, Weinstein EG, Bartel DP (2001) An abundant class of tiny RNAs with probable regulatory roles in *Caenorhabditis elegans* . Science 294: 858–862. 11679671 10.1126/science.1065062

[26] Lim LP, Lau NC, Weinstein EG, Abdelhakim A, Yekta S, et al (2003) The microRNAs of *Caenorhabditis elegans* . Genes Dev 17: 991–1008. 12672692 10.1101/gad.1074403 PMC196042

[27] Ambros V, Lee RC, Lavanway A, Williams PT, Jewell D (2003) MicroRNAs and other tiny endogenous RNAs in *C. elegans* . Curr Biol 13: 807–818. 12747828 10.1016/s0960-9822(03)00287-2

[28] Grad Y, Aach J, Hayes GD, Reinhart BJ, Church GM, et al (2003) Computational and experimental identification of *C. elegans* microRNAs. Mol Cell 11: 1253–1263. 12769849 10.1016/s1097-2765(03)00153-9

[29] Ruby JG, Jan C, Player C, Axtell MJ, Lee W, et al (2006) Large-scale sequencing reveals 21U-RNAs and additional microRNAs and endogenous siRNAs in *C. elegans* . Cell 127: 1193–1207. 17174894 10.1016/j.cell.2006.10.040

[30] Kato M, de Lencastre A, Pincus Z, Slack FJ (2009) Dynamic expression of small non-coding RNAs, including novel microRNAs and piRNAs/21U-RNAs, during *Caenorhabditis elegans* development. Genome Biol 10: R54. 19460142 10.1186/gb-2009-10-5-r54 PMC2718520

[31] Zisoulis DG, Lovci MT, Wilbert ML, Hutt KR, Liang TY, et al (2010) Comprehensive discovery of endogenous Argonaute binding sites in *Caenorhabditis elegans* . Nat Struct Mol Biol 17: 173–179. 20062054 10.1038/nsmb.1745 PMC2834287

[32] Poole CB, Davis PJ, Jin J, McReynolds LA (2010) Cloning and bioinformatic identification of small RNAs in the filarial nematode, *Brugia malayi* . Mol Biochem Parasitol 169: 87–94. 19874857 10.1016/j.molbiopara.2009.10.004

[33] Winter AD, Weir W, Hunt M, Berriman M, Gilleard JS, et al (2012) Diversity in parasitic nematode genomes: the microRNAs of Brugia pahangi and Haemonchus contortus are largely novel. BMC Genomics 13: 4. 22216965 10.1186/1471-2164-13-4 PMC3282659

[34] Pak J, Fire A (2007) Distinct populations of primary and secondary effectors during RNAi in *C. elegans* . Science 315: 241–244. 17124291 10.1126/science.1132839

[35] Sijen T, Steiner FA, Thijssen KL, Plasterk RH (2007) Secondary siRNAs result from unprimed RNA synthesis and form a distinct class. Science 315: 244–247. 17158288 10.1126/science.1136699

[36] Gu W, Shirayama M, Conte D Jr, Vasale J, Batista PJ, et al (2009) Distinct argonaute-mediated 22G-RNA pathways direct genome surveillance in the *C. elegans* germline. Mol Cell 36: 231–244. 19800275 10.1016/j.molcel.2009.09.020 PMC2776052

[37] Basyuk E, Suavet F, Doglio A, Bordonne R, Bertrand E (2003) Human let-7 stem-loop precursors harbor features of RNase III cleavage products. Nucleic Acids Res 31: 6593–6597. 14602919 10.1093/nar/gkg855 PMC275551

[38] Carmell MA, Hannon GJ (2004) RNase III enzymes and the initiation of gene silencing. Nat Struct Mol Biol 11: 214–218. 14983173 10.1038/nsmb729

[39] Ambros V, Bartel B, Bartel DP, Burge CB, Carrington JC, et al (2003) A uniform system for microRNA annotation. RNA 9: 277–279. 12592000 10.1261/rna.2183803 PMC1370393

[40] Ibanez-Ventoso C, Vora M, Driscoll M (2008) Sequence relationships among *C. elegans*, *D. melanogaster* and human microRNAs highlight the extensive conservation of microRNAs in biology. PLoS One 3: e2818. 18665242 10.1371/journal.pone.0002818 PMC2486268

[41] McReynolds LA, DeSimone SM, Williams SA (1986) Cloning and comparison of repeated DNA sequences from the human filarial parasite *Brugia malayi* and the animal parasite *Brugia pahangi* . Proc Natl Acad Sci USA 83: 797–801. 3003750 10.1073/pnas.83.3.797 PMC322952

[42] Friedlander MR, Chen W, Adamidi C, Maaskola J, Einspanier R, et al (2008) Discovering microRNAs from deep sequencing data using miRDeep. Nat Biotechnol 26: 407–415. 18392026 10.1038/nbt1394

[43] Jin J, Cid M, Poole CB, McReynolds LA (2010) Protein mediated miRNA detection and siRNA enrichment using p19. Biotechniques 48: : xvii–xxiii. 10.2144/000113364 20569217

[44] Wanunu M, Dadosh T, Ray V, Jin J, McReynolds L, et al (2010) Rapid electronic detection of probe-specific microRNAs using thin nanopore sensors. Nature nanotechnology 5: 807–814. 10.1038/nnano.2010.202 20972437

[45] Labib M, Khan N, Ghobadloo SM, Cheng J, Pezacki JP, et al (2013) Three-mode electrochemical sensing of ultralow microRNA levels. Journal of the American Chemical Society 135: 3027–3038. 23362834 10.1021/ja308216z

[46] Wang J, Czech B, Crunk A, Wallace A, Mitreva M, et al (2011) Deep small RNA sequencing from the nematode Ascaris reveals conservation, functional diversification, and novel developmental profiles. Genome research 21: 1462–1477. 21685128 10.1101/gr.121426.111 PMC3166831

[47] Sapio MR, Hilliard MA, Cermola M, Favre R, Bazzicalupo P (2005) The Zona Pellucida domain containing proteins, CUT-1, CUT-3 and CUT-5, play essential roles in the development of the larval alae in *Caenorhabditis elegans* . Dev Biol 282: 231–245. 15936343 10.1016/j.ydbio.2005.03.011

[48] Hussain M, Frentiu FD, Moreira LA, O'Neill SL, Asgari S (2011) *Wolbachia* uses host microRNAs to manipulate host gene expression and facilitate colonization of the dengue vector *Aedes aegypti* . Proc Natl Acad Sci U S A 108: 9250–9255. 21576469 10.1073/pnas.1105469108 PMC3107320

[49] Griffiths-Jones S, Grocock RJ, van Dongen S, Bateman A, Enright AJ (2006) miRBase: microRNA sequences, targets and gene nomenclature. Nucleic Acids Res 34: D140–144. 16381832 10.1093/nar/gkj112 PMC1347474

[50] Griffiths-Jones S, Saini HK, van Dongen S, Enright AJ (2008) miRBase: tools for microRNA genomics. Nucleic Acids Res 36: D154–158. 17991681 10.1093/nar/gkm952 PMC2238936

[51] Miska EA, Alvarez-Saavedra E, Abbott AL, Lau NC, Hellman AB, et al (2007) Most *Caenorhabditis elegans* microRNAs are individually not essential for development or viability. PLoS Genet 3: e215. 18085825 10.1371/journal.pgen.0030215 PMC2134938

[52] Alvarez-Saavedra E, Horvitz HR (2010) Many families of *C. elegans* microRNAs are not essential for development or viability. Curr Biol 20: 367–373. 20096582 10.1016/j.cub.2009.12.051 PMC2844791

[53] Leaman D, Chen PY, Fak J, Yalcin A, Pearce M, et al (2005) Antisense-mediated depletion reveals essential and specific functions of microRNAs in *Drosophila* development. Cell 121: 1097–1108. 15989958 10.1016/j.cell.2005.04.016

[54] Conradt B, Horvitz HR (1998) The *C. elegans* protein EGL-1 is required for programmed cell death and interacts with the Bcl-2-like protein CED-9. Cell 93: 519–529. 9604928 10.1016/s0092-8674(00)81182-4

[55] Zhang L, Hammell M, Kudlow BA, Ambros V, Han M (2009) Systematic analysis of dynamic miRNA-target interactions during C. elegans development. Development 136: 3043–3055. 19675127 10.1242/dev.039008 PMC2730362

[56] Hafner M, Renwick N, Brown M, Mihailovic A, Holoch D, et al (2011) RNA-ligase-dependent biases in miRNA representation in deep-sequenced small RNA cDNA libraries. RNA 17: 1697–1712. 21775473 10.1261/rna.2799511 PMC3162335

[57] Zhuang F, Fuchs RT, Sun Z, Zheng Y, Robb GB (2012) Structural bias in T4 RNA ligase-mediated 3′-adapter ligation. Nucleic acids research 40: e54. 22241775 10.1093/nar/gkr1263 PMC3326334

[58] Isik M, Korswagen HC, Berezikov E (2010) Expression patterns of intronic microRNAs in *Caenorhabditis elegans* . Silence 1: 5. 20226079 10.1186/1758-907X-1-5 PMC2835999

[59] de Lencastre A, Pincus Z, Zhou K, Kato M, Lee SS, et al (2010) MicroRNAs both promote and antagonize longevity in *C. elegans* . Curr Biol 20: 2159–2168. 21129974 10.1016/j.cub.2010.11.015 PMC3023310

[60] Boulias K, Horvitz HR (2012) The C. elegans microRNA mir-71 acts in neurons to promote germline-mediated longevity through regulation of DAF-16/FOXO. Cell metabolism 15: 439–450. 22482727 10.1016/j.cmet.2012.02.014 PMC3344382

[61] Karp X, Hammell M, Ow MC, Ambros V (2011) Effect of life history on microRNA expression during *C. elegans* development. RNA 17: 639–651. 21343388 10.1261/rna.2310111 PMC3062175

[62] Ponnudurai T, Denham DA, Rogers R (1975) Studies on *Brugia pahangi* 9. The longevity of microfilariae transfused from cat to cat. J Helminthol 49: 25–30. 1127214

[63] Le Bechec A, Portales-Casamar E, Vetter G, Moes M, Zindy PJ, et al (2011) MIR@NT@N: a framework integrating transcription factors, microRNAs and their targets to identify sub-network motifs in a meta-regulation network model. BMC Bioinformatics 12: 67. 21375730 10.1186/1471-2105-12-67 PMC3061897

[64] Shalgi R, Lieber D, Oren M, Pilpel Y (2007) Global and local architecture of the mammalian microRNA-transcription factor regulatory network. PLoS Comput Biol 3: e131. 17630826 10.1371/journal.pcbi.0030131 PMC1914371

[65] Martinez NJ, Ow MC, Barrasa MI, Hammell M, Sequerra R, et al (2008) A *C. elegans* genome-scale microRNA network contains composite feedback motifs with high flux capacity. Genes Dev 22: 2535–2549. 18794350 10.1101/gad.1678608 PMC2546694

[66] Castanotto D, Rossi JJ (2009) The promises and pitfalls of RNA-interference-based therapeutics. Nature 457: 426–433. 19158789 10.1038/nature07758 PMC2702667

[67] Krutzfeldt J, Rajewsky N, Braich R, Rajeev KG, Tuschl T, et al (2005) Silencing of microRNAs in vivo with ‘antagomirs’. Nature 438: 685–689. 16258535 10.1038/nature04303

[68] Obad S, dos Santos CO, Petri A, Heidenblad M, Broom O, et al (2011) Silencing of microRNA families by seed-targeting tiny LNAs. Nat Genet 43: 371–378. 21423181 10.1038/ng.786 PMC3541685

[69] Weese D, Emde AK, Rausch T, Doring A, Reinert K (2009) RazerS–fast read mapping with sensitivity control. Genome Res 19: 1646–1654. 19592482 10.1101/gr.088823.108 PMC2752123

[70] Zhang H, Kolb FA, Jaskiewicz L, Westhof E, Filipowicz W (2004) Single processing center models for human Dicer and bacterial RNase III. Cell 118: 57–68. 15242644 10.1016/j.cell.2004.06.017

[71] Cock PJ, Antao T, Chang JT, Chapman BA, Cox CJ, et al (2009) Biopython: freely available Python tools for computational molecular biology and bioinformatics. Bioinformatics 25: 1422–1423. 19304878 10.1093/bioinformatics/btp163 PMC2682512

[72] Katoh K, Toh H (2008) Improved accuracy of multiple ncRNA alignment by incorporating structural information into a MAFFT-based framework. BMC Bioinformatics 9: 212. 18439255 10.1186/1471-2105-9-212 PMC2387179

[73] Huelsenbeck JP, Ronquist F (2001) MRBAYES: Bayesian inference of phylogenetic trees. Bioinformatics 17: 754–755. 11524383 10.1093/bioinformatics/17.8.754

[74] Dereeper A, Guignon V, Blanc G, Audic S, Buffet S, et al (2008) Phylogeny.fr: robust phylogenetic analysis for the non-specialist. Nucleic Acids Res 36: W465–469. 18424797 10.1093/nar/gkn180 PMC2447785

[75] Shi R, Chiang VL (2005) Facile means for quantifying microRNA expression by real-time PCR. Biotechniques 39: 519–525. 16235564 10.2144/000112010

