# Supplementary figures and images for: Diversity and Expression of MicroRNAs in the Filarial Parasite, Brugia malayi
Source: PLoS One. 2014 May 13;9(5):e96498. doi: 10.1371/journal.pone.0096498 (PMC4019659; doi:10.1371/journal.pone.0096498)

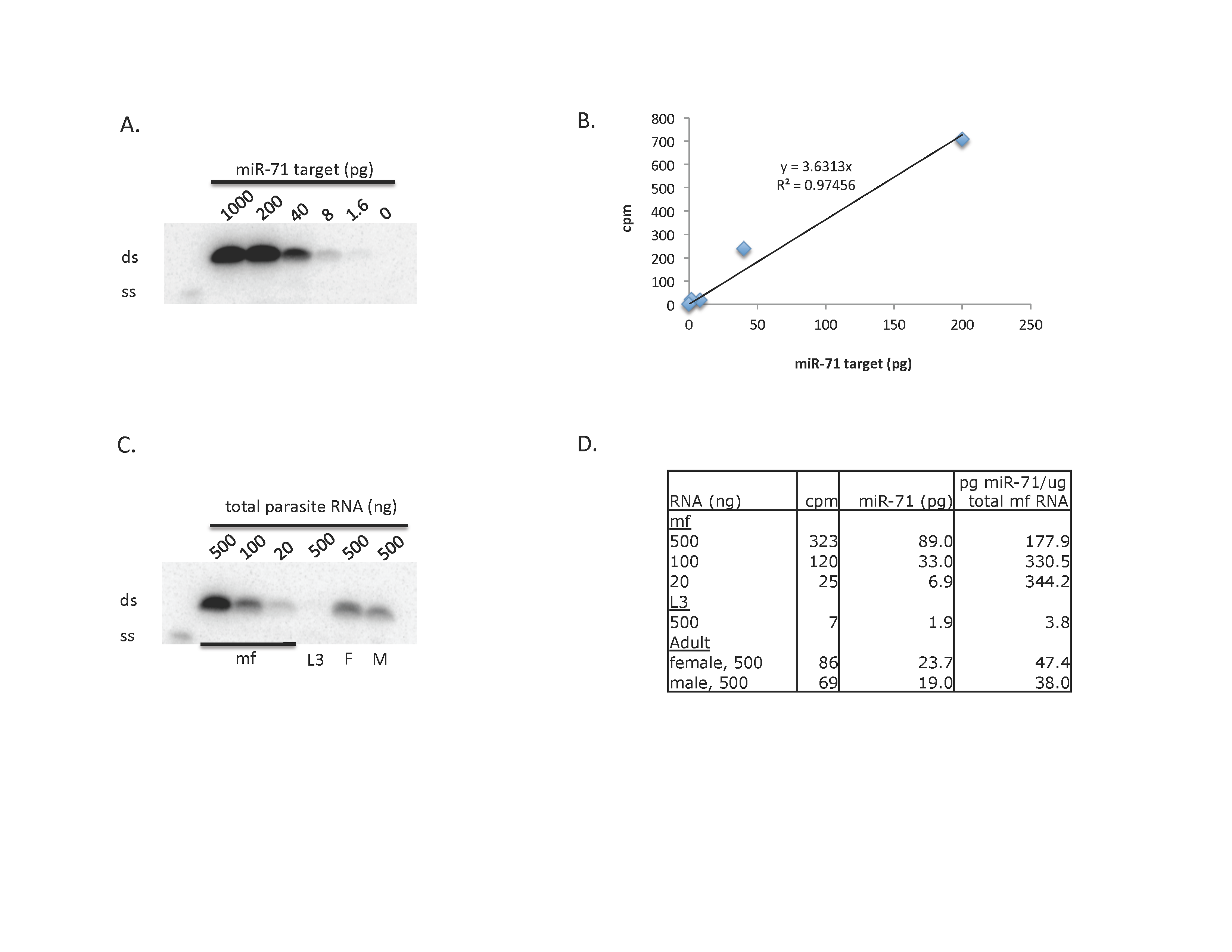

Supplement: Figure S1 — Quantitation of miR-71 in B. malayi using p19. A standard curve was generated by hybridizing defined quantities of synthetic B. malayi miR-71 with 1 ng of 32P-labeled miR-71 probe as described [43]. (A) Autoradiograph of a 20% non-denaturing gel showing the miR-71:probe hybrid eluted from p19 beads. The mobility of the double-stranded (ds) miRNA:RNA probe duplex and single-stranded (ss) RNA probe is shown to the right of the gel. (B) The amount of radioactivity in 10 µl (1/5th) of the eluted miR-71: probe hybrid was measured by scintillation counting and plotted against known amounts of synthetic miR-71 to generate a standard curve. (C) Autoradiograph of a non-denaturing gel showing p19 eluants of miR-71 from RNA samples of B. malayi males (M), females (F), microfilariae (mf) and third stage larvae (L3). The position of the double-stranded (ds) miRNA:RNA probe duplex and single-stranded (ss) RNA probe is shown to the right of the gel. (D) The quantity of miR-71 in the different stages of B. malayi was calculated from the standard curve using the amount of radioactivity in 10 µl of p19 eluant. (TIFF) [file pone.0096498.s001.tiff]

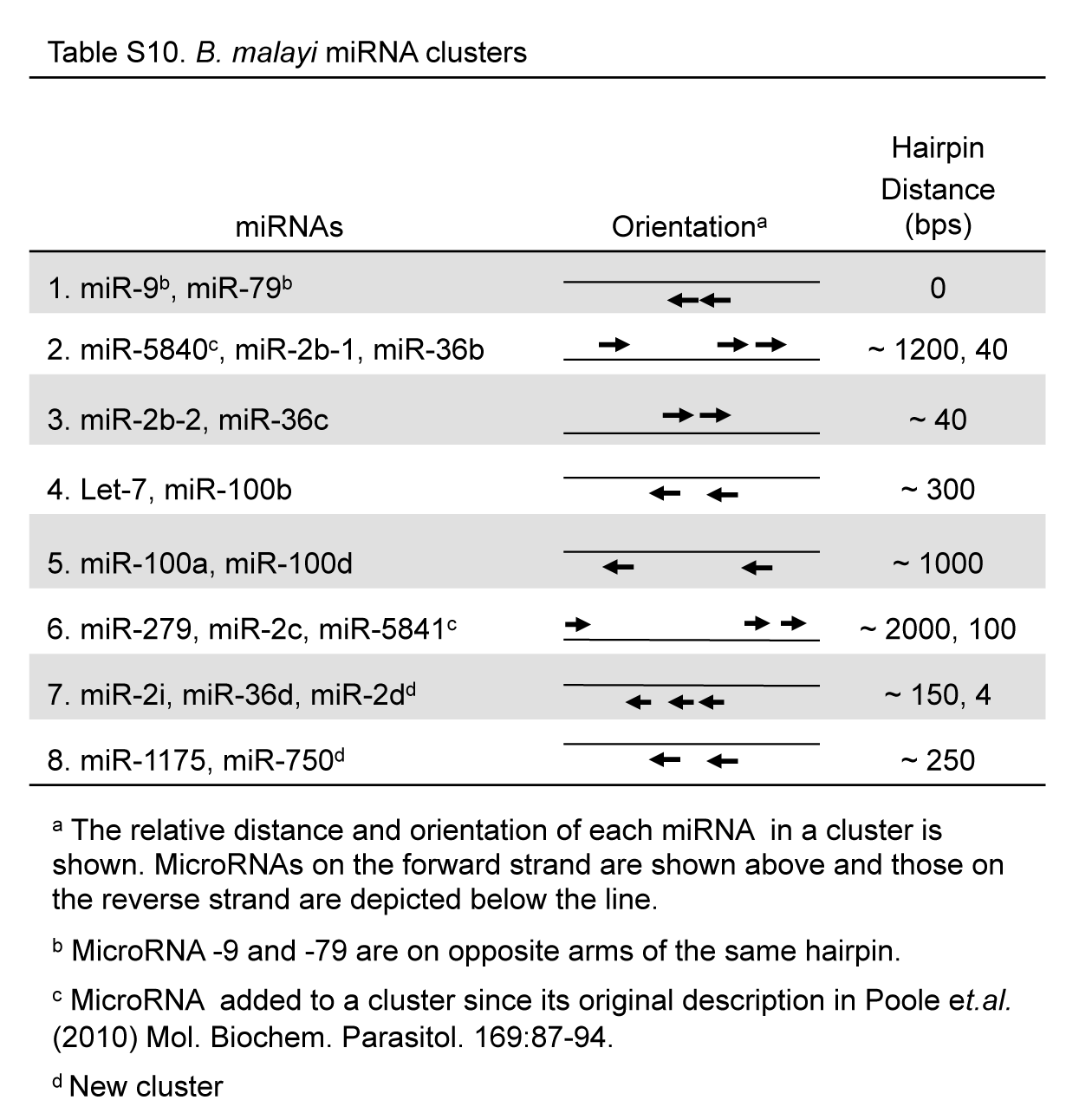

Supplement: Table S10 — B. malayi miRNA clusters. The table lists the miRNAs that are clustered in the B. malayi genome. The orientation (forward or reverse strand) and relative position of the miRNAs in a cluster is based on the annotated B. malayi genome. The bp distance between the miRNAs in a cluster is shown in the right hand column. MicroRNA-9 and miR-79 are on opposite arms of the same hairpin. (XLSX) [file pone.0096498.s011.xlsx]
